# Clinicopathological characteristics across the HER2 expression spectrum in invasive breast carcinoma of no special type: a single-centre retrospective study

**DOI:** 10.1186/s13000-026-01791-x

**Published:** 2026-05-21

**Authors:** Aditi Tiwary, Sachin Sebastian Francis, Swati Sharma, Ananth Pai

**Affiliations:** 1 Manipal Academy of Higher Education, Manipal, India; 2 Institute of Liver and Biliary Sciences, New Delhi, India

**Keywords:** HER2-low breast cancer, HER2-ultralow, Invasive breast carcinoma of no special type, Ki-67 proliferation index, Stromal tumour-infiltrating lymphocytes

## Abstract

**Background:**

The efficacy of trastuzumab deruxtecan in HER2-low metastatic breast cancer has shifted HER2 classification from a binary framework toward a continuous expression spectrum that now includes HER2-low and HER2-ultralow categories. Accurate characterization of these subgroups is essential for therapeutic stratification, yet their clinicopathological profiles remain incompletely defined, particularly in the Indian population. This study compared the clinicopathological features and short-term outcomes of invasive breast carcinoma of no special type across four HER2-defined subgroups.

**Methods:**

Three hundred cases of invasive breast carcinoma of no special type diagnosed on core needle biopsy at a tertiary centre in southern India (2022–2023) were classified into HER2-positive, HER2-low, HER2-ultralow, and HER2-negative subgroups using immunohistochemistry with fluorescence in situ hybridisation confirmation. Associations were evaluated using chi-square tests, Kruskal-Wallis tests, binary logistic regression, correspondence analysis, and Kaplan-Meier survival analysis.

**Results:**

HER2-low constituted the largest subgroup at 51.7%, followed by HER2-positive (32.3%), HER2-ultralow (9.0%), and HER2-negative (7.0%). HER2-low tumours demonstrated significantly higher oestrogen receptor (74.8%) and progesterone receptor (61.3%) positivity, lower Nottingham grade, and a greater proportion of low Ki-67 proliferation index compared to the other subgroups. Age, menopausal status, clinical stage, tumour size, lymphovascular invasion, perineural invasion, and stromal tumour-infiltrating lymphocyte density did not differ significantly across the four subgroups. On multivariate logistic regression, progesterone receptor positivity was the sole independent predictor of HER2-low status (odds ratio 1.92, 95% confidence interval 1.05–3.50, *p* = 0.034). The triple-negative phenotype increased progressively from HER2-low (25.2%) through HER2-ultralow (40.7%) to HER2-negative (57.1%). Correspondence analysis placed HER2-ultralow in an intermediate position between HER2-low and HER2-negative. No significant survival differences were observed over a median follow-up of 23 months.

**Conclusions:**

HER2-low tumours represent the majority of invasive breast carcinomas in this Indian cohort and exhibit a distinct profile defined by hormone receptor enrichment, lower grade, and reduced proliferative activity. The intermediate phenotype of HER2-ultralow supports its recognition as a biologically separate category. These findings highlight the importance of standardised HER2 reporting across the full expression spectrum to guide antibody-drug conjugate therapy eligibility.

## Introduction

Breast cancer remains the most commonly diagnosed malignancy among women worldwide and a leading cause of cancer-related mortality. It accounts for approximately 2.3 million new cases and over 685,000 deaths globally, making it the most frequently diagnosed cancer across both developed and developing regions [1]. In India, breast cancer has emerged as the most common malignancy among women in several urban cancer registries, with a steadily increasing incidence over the past two decades [2]. Despite advances in screening strategies, surgical techniques, and systemic therapies, breast cancer continues to demonstrate considerable biological heterogeneity, which remains a major determinant of disease behaviour, treatment response, and clinical outcome.

Histopathological evaluation, together with immunohistochemical assessment of predictive biomarkers, forms the cornerstone of diagnostic classification and therapeutic stratification in invasive breast carcinoma (IBC). Among these biomarkers, the human epidermal growth factor receptor 2 (HER2), encoded by the *ERBB2* gene, occupies a central role in breast cancer biology. HER2 overexpression or gene amplification is observed in approximately 15–20% of invasive breast cancers and is associated with increased proliferative activity, aggressive tumour behaviour, and a historically poorer prognosis [3]. The advent of HER2-targeted therapies, particularly trastuzumab, has substantially improved survival outcomes in HER2-positive breast cancer and has transformed HER2 from a purely prognostic marker into one of the most clinically actionable therapeutic targets in oncology [4].

Traditionally, IBCs have been classified as either HER2-positive, HER2-equivocal or HER2-negative based on immunohistochemical (IHC) expression followed by a reflex in situ hybridization (ISH) testing in equivocal cases. Tumours demonstrating strong circumferential membranous staining (IHC 3+) and/or gene amplification on fluorescence in situ hybridization (FISH) are categorized as HER2-positive, while all remaining tumours were generally considered HER2-negative. However, a substantial proportion of tumours previously grouped within the HER2-negative category demonstrate low levels of HER2 protein expression, typically defined as IHC 1 + or IHC 2 + without gene amplification. These tumours have recently been designated as HER2-low breast cancers, representing a clinically and biologically heterogeneous subset that may respond to novel HER2-directed therapies [5].

The clinical significance of HER2-low tumours gained considerable attention following the DESTINY-Breast04 trial, which demonstrated a significant survival benefit with trastuzumab deruxtecan (T-DXd) in patients with previously treated metastatic HER2-low breast cancer [6]. These findings have fundamentally altered the therapeutic landscape of breast cancer by expanding the population of patients who may benefit from HER2-targeted antibody–drug conjugates (ADCs). More recently, interest has extended even further toward the lower end of the HER2 expression spectrum with the emergence of the HER2-ultralow category. HER2-ultralow tumours demonstrate faint, incomplete membranous staining in a very small proportion of tumour cells that would previously have been classified as HER2-negative. Preliminary data suggest that these tumours may possess distinct clinicopathological characteristics and could potentially benefit from emerging HER2-directed therapies [7].

The recognition of HER2-low and HER2-ultralow categories therefore represents an important conceptual shift in the classification of breast cancer. HER2 expression is increasingly being viewed as a spectrum encompassing HER2-positive, HER2-low, HER2-ultralow, and HER2-negative tumours. This shift has significant diagnostic implications, as subtle differences in staining intensity and distribution can now influence treatment eligibility. Emerging studies have also suggested that tumours across the HER2 expression continuum may exhibit differences in clinicopathological features, hormone receptor expression, proliferative indices, and tumour microenvironment characteristics [8, 9]. Nevertheless, the biological behaviour and clinical implications of these emerging categories remain incompletely understood, particularly in Asian and Indian populations where available data remain limited.

In this context, a comprehensive evaluation of invasive breast carcinomas across the HER2 expression continuum is warranted. The present study therefore examines invasive breast carcinoma of no special type (IBC-NST) across four HER2-defined subgroups: HER2-positive, HER2-low, HER2-ultralow, and HER2-negative. By correlating HER2 expression with clinicodemographic parameters, histologic grade, hormone receptor status, Ki-67 proliferation index, stromal tumour-infiltrating lymphocytes (sTILs), and molecular subtype, we aim to characterize the clinicopathological features associated with these emerging HER2 categories. Through this analysis, the study seeks to contribute to the evolving understanding of HER2-defined subtypes and their implications in contemporary diagnostic practice.

## Materials and methods

### Study design and case selection

This retrospective observational study was conducted in the Department of Pathology at Kasturba Medical College, Manipal Academy of Higher Education (MAHE), Manipal, India. The study included all eligible cases diagnosed between 1 January 2022 and 31 December 2023. The study protocol was reviewed and approved by the Institutional Ethics Committee (IEC2: 93/2023).

All cases diagnosed as invasive breast carcinoma, no special type (IBC-NST), on core needle biopsy during the study period were considered for inclusion. Cases were excluded if histopathological slides or paraffin-embedded tissue blocks were unavailable for review, if immunohistochemistry (IHC) had not been performed, or if IHC slides could not be retrieved from the institutional archives. Cases lacking essential clinicopathological information or without available fluorescence in situ hybridization (FISH) reports for HER2-equivocal tumours were also excluded. Following application of these criteria, a total of 300 cases were included in the final analysis.

### Data collection

Demographic and clinical data were retrieved from institutional electronic records including the Laboratory Information System (LIS), Radiology Information System/PACS (RIS-PACS), and electronic medical records. Extracted variables included age, sex, menopausal status, parity, presenting symptoms, family history of breast cancer, radiological findings, clinical stage at presentation, treatment modality, and available follow-up information. For patients who subsequently underwent surgical excision in the form of lumpectomy or mastectomy, additional pathological parameters such as tumour size, focality, nodal metastasis, margin status, and residual disease following neoadjuvant chemotherapy (NACT) were obtained from corresponding histopathology reports.

### Histopathological evaluation

Archived haematoxylin and eosin (H&E) slides were retrieved and reviewed. Histologic evaluation included determination of Nottingham histologic grade using the modified Scarff-Bloom-Richardson scoring system, based on tubule formation, nuclear pleomorphism, and mitotic activity. Additional morphological parameters assessed included lymphovascular invasion (LVI), perineural invasion (PNI), tumour necrosis, and stromal tumour-infiltrating lymphocytes (sTILs). sTILs were assessed in accordance with the recommendations of the International TILs Working Group and recorded as the percentage of stromal area occupied by mononuclear inflammatory cells within the tumour microenvironment. For analytical purposes, sTIL density was categorized as low (< 10%), moderate (10–50%), or high (> 50%).

Immunohistochemical slides were retrieved and re-evaluated to ensure uniform interpretation of biomarker expression across the cohort. The biomarkers assessed included estrogen receptor (ER; clone EP1), progesterone receptor (PR; clone PgR636), HER2 (clone c-erbB2), and Ki-67 (clone MIB-1). Hormone receptor expression was interpreted according to current ASCO/CAP guidelines. The Ki-67 proliferation index was assessed in tumour hotspots by counting a minimum of 500 tumour cells, and the proportion of positively stained nuclei was recorded as a percentage. For categorical analyses, a clinically relevant threshold of 20% was used to classify tumours as low proliferative (< 20%) or high proliferative (≥ 20%).

### HER2 assessment and subgroup classification

HER2 expression was evaluated using immunohistochemistry based on CAP biomarker reporting protocol 2025 update and ASCO/CAP guidelines [10] and correlated with FISH results in equivocal cases. Tumours were subsequently stratified across the HER2 expression spectrum using a stepwise classification approach. Cases demonstrating an IHC score of 3 + or those with IHC 2 + staining and confirmed ERBB2 amplification on FISH were classified as HER2-positive. Tumours with IHC 1 + staining, or IHC 2 + staining without gene amplification on FISH, were categorized as HER2-low. Among cases scored as IHC 0, those demonstrating any perceptible faint, incomplete membranous staining in ≤ 10% of tumour cells were classified as HER2-ultralow, while tumours with complete absence of any membranous staining were categorized as HER2-negative. This distinction was made jointly by two pathologists (AT and SS) who independently reviewed all IHC 0 cases; discordant interpretations were resolved by consensus at a multiheaded microscope.

Molecular subtype classification was approximated using surrogate immunohistochemical profiles incorporating ER, PR, HER2, and Ki-67 expression. Based on these markers, tumours were categorized into Luminal A, Luminal B, HER2-enriched, and triple-negative molecular subtypes.

### Statistical analysis

Statistical analyses were performed using IBM SPSS Statistics version 21.0. Continuous variables were initially assessed for normality using the Shapiro–Wilk test. Normally distributed variables were summarized as mean ± standard deviation, whereas non-normally distributed variables were presented as median with interquartile range (IQR). Comparisons between HER2 subgroups were performed using one-way analysis of variance (ANOVA) for normally distributed continuous variables and the Kruskal–Wallis test for non-parametric variables. Associations between categorical variables were evaluated using Pearson’s chi-square test or Fisher’s exact test where appropriate. Effect sizes for categorical associations were estimated using Cramer’s V.

To explore whether the continuous Ki-67 distributions differed in shape (and not merely in central tendency), pairwise two-sample Kolmogorov–Smirnov tests were performed between selected HER2 subgroup pairs. Correlations between continuous variables, including Ki-67, age, and sTIL percentage, were evaluated using Spearman rank correlation analysis. To identify clinicopathological variables independently associated with HER2-low tumours, binary logistic regression analysis was performed. Regression models included relevant variables such as hormone receptor status, Ki-67 category, sTIL category, histologic grade, and molecular subtype. Model adequacy was assessed using the Hosmer–Lemeshow goodness-of-fit test, and explanatory power was evaluated using the Nagelkerke R² statistic.

To further explore the multivariate relationships between HER2 subgroups and clinicopathological variables, correspondence analysis was performed, allowing visualization of associations across categorical variables within a multidimensional framework.

Follow-up data including recurrence, mortality, and disease-free survival were assessed. Survival distributions were estimated using Kaplan–Meier analysis, and differences between HER2 subgroups were compared using the log-rank (Mantel–Cox) test. A two-sided p-value ≤ 0.05 was considered statistically significant for all analyses.

## Results

**Fig. 1:**
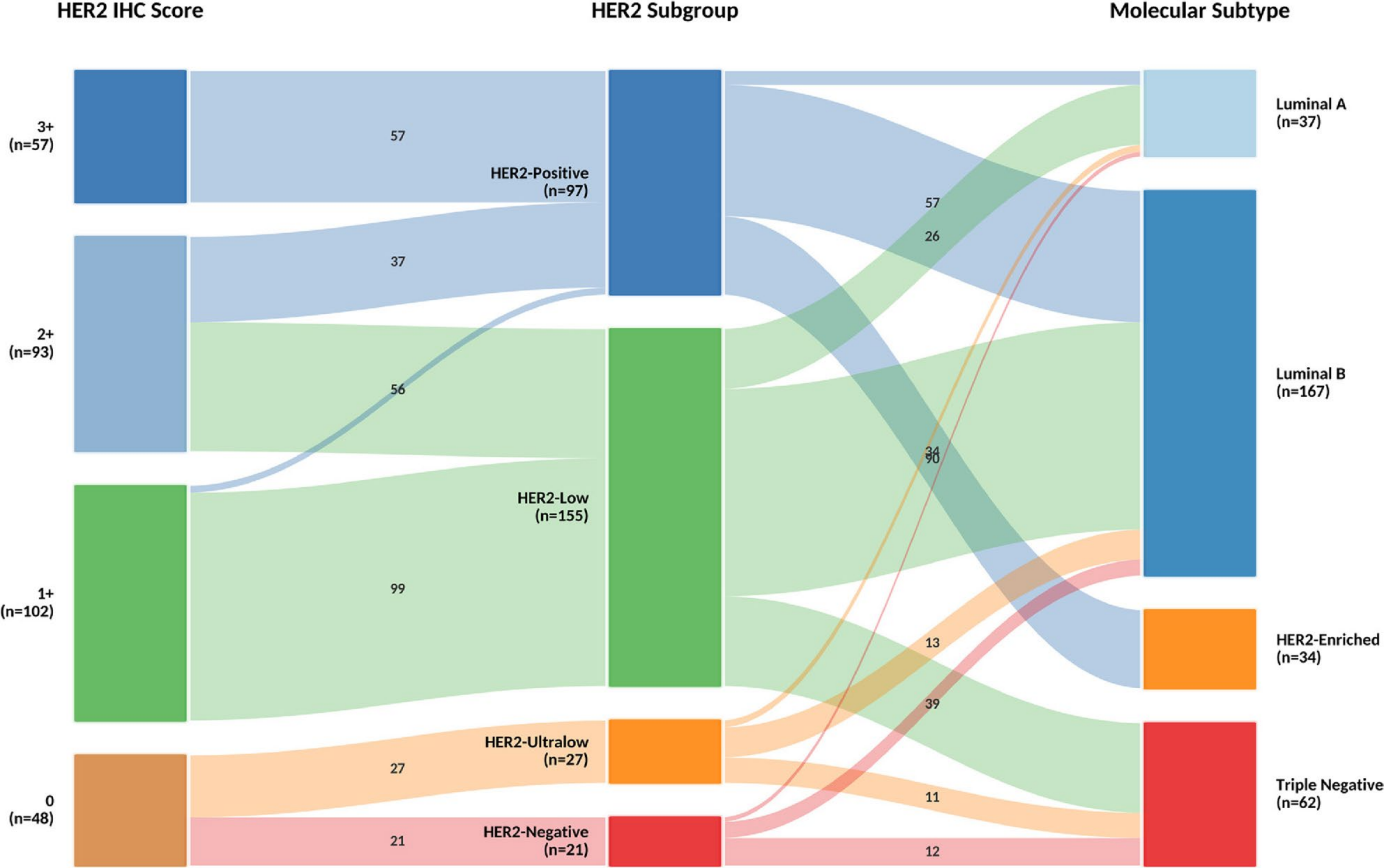
Sankey diagram showing the classification cascade from HER2 IHC score through HER2 expression subgroup to molecular subtype in 300 IBC-NST cases. Three-column flow diagram in which vertical bar heights are proportional to case counts and flow widths represent the number of cases transitioning between categories. Left column: HER2 IHC scores, ordered from top to bottom as 3+, 2+, 1+, and 0. Centre column: HER2 expression subgroups defined by integration of IHC and FISH results, ordered consistently as HER2-Positive, HER2-Low, HER2-Ultralow, and HER2-Negative from top to bottom. Right column: surrogate molecular subtypes (Luminal A, Luminal B, HER2-Enriched, Triple Negative). Colour coding follows HER2 subgroup assignment: blue = HER2-Positive, green = HER2-Low, orange = HER2-Ultralow, red = HER2-Negative. Case counts are annotated on each flow path

A total of 300 cases of IBC-NST were stratified into four groups based on HER2 IHC scoring and FISH confirmation: HER2-positive (*n* = 97, 32.3%; 95% CI: 27.1–37.9%), HER2-low (*n* = 155, 51.7%; 95% CI: 45.9–57.4%), HER2-ultralow (*n* = 27, 9.0%; 95% CI: 6.0–12.8%), and HER2-negative (*n* = 21, 7.0%; 95% CI: 4.4–10.5%). The classification cascade from IHC score through FISH-determined subgroup to molecular subtype is illustrated in Fig. 1. Among IHC 2 + cases (*n* = 93), 37 (39.8%) were reclassified as HER2-positive after FISH amplification was confirmed, while 56 (60.2%) entered the HER2-low pathway as FISH non-amplified. All IHC 1 + cases (*n* = 99) mapped exclusively to HER2-low, constituting 63.9% of the HER2-low subgroup.

Demographic and clinical characteristics are presented in Table 1. The mean age of the cohort was 52.6 ± 11.6 years, with similar distributions across HER2 groups (one-way ANOVA: F(3,296) = 1.229, *p* = 0.299). Most patients were postmenopausal (62.7%) and presented with a breast lump (99.0%). Tumours were predominantly unifocal (71.3%) and measured 2–5 cm (57.3%). Clinical stage, tumour size, laterality, and treatment modality did not differ significantly across HER2 subgroups. Parity showed borderline significance (χ² = 7.98, *p* = 0.046), with a higher proportion of nulliparity observed in HER2-negative tumours (28.6%) compared to the other groups.

**Table 1:** Baseline demographic and clinical characteristics by HER2 expression subgroup

Variable	Overall (*n* = 300)	HER2-Positive (*n* = 97)	HER2-Low (*n* = 155)	HER2-Ultralow (*n* = 27)	HER2-Negative (*n* = 21)	*p*-value
Age (years), mean ± SD	52.6 ± 11.6	51.6 ± 10.7	53.8 ± 11.8	52.4 ± 13.8	49.6 ± 11.4	0.299^a^
Menopausal status						0.128^b^
Premenopausal	112 (37.3)	42 (43.3)	48 (31.0)	12 (44.4)	10 (47.6)	
Postmenopausal	188 (62.7)	55 (56.7)	107 (69.0)	15 (55.6)	11 (52.4)	
Parity^d^						**0.046** ^b^
Multiparous	254 (89.4)	89 (91.8)	126 (90.6)	24 (88.9)	15 (71.4)	
Nulliparous	30 (10.6)	8 (8.2)	13 (9.4)	3 (11.1)	6 (28.6)	
Obesity^d^						0.421^b^
Yes	75 (33.6)	30 (40.5)	33 (29.5)	7 (36.8)	5 (27.8)	
No	148 (66.4)	44 (59.5)	79 (70.5)	12 (63.2)	13 (72.2)	
Clinical stage^d^						0.425^b^
I	20 (6.7)	2 (2.1)	14 (9.2)	2 (7.4)	2 (9.5)	
II	128 (43.1)	44 (45.8)	69 (45.4)	7 (25.9)	8 (38.1)	
III	82 (27.6)	28 (29.2)	38 (25.0)	10 (37.0)	6 (28.6)	
IV	67 (22.6)	22 (22.9)	32 (21.1)	8 (29.6)	5 (23.8)	
Tumour size^d^						0.720^b^
<2 cm	55 (18.7)	17 (18.1)	29 (19.1)	5 (18.5)	4 (19.0)	
2–5 cm	172 (58.5)	52 (55.3)	95 (62.5)	14 (51.9)	11 (52.4)	
>5 cm	67 (22.8)	25 (26.6)	28 (18.4)	8 (29.6)	6 (28.6)	

^a^One-Way ANOVA. ^b^Pearson’s chi-square test (Fisher’s exact when > 20% cells had expected count < 5).  ^d^Percentages calculated after excluding missing data. NACT, neoadjuvant chemotherapy. 95% Clopper-Pearson CIs for subgroup incidence: HER2-Positive 27.1–37.9%; HER2-Low 45.9–57.4%; HER2-Ultralow 6.0–12.8%; HER2-Negative 4.4–10.5%

Significant *p*-values are highlighted in bold

Histopathological features and biomarker profiles are summarized in Tables 2 and 3. Nottingham histologic grade differed significantly across subgroups (χ² = 22.67, *p* < 0.001, Cramer’s V = 0.194). HER2-low tumours were predominantly grade 2 (55.5%), with the highest proportion of grade 1 tumours among all subgroups (22.6%). In contrast, grade 3 morphology predominated in HER2-negative (52.4%) and HER2-ultralow (40.7%) tumours. Lymphovascular invasion (26.7%), perineural invasion (6.0%), and tumour necrosis (38.0%) were evenly distributed across groups and showed no significant association with HER2 status. Hormone receptor status was significantly associated with HER2 subgroup. ER positivity was highest in HER2-low tumours (74.8%) and lowest in HER2-negative (38.1%; χ² = 13.45, *p* = 0.004, Cramer’s V = 0.212). PR positivity showed the most pronounced separation across the spectrum: 61.3% in HER2-low versus 35.1% in HER2-positive, 55.6% in HER2-ultralow, and 23.8% in HER2-negative (χ² = 22.66, *p* < 0.001, V = 0.275).

**Table 2:** Histopathological features, biomarker profile, and molecular subtype by HER2 expression subgroup

Variable	Overall (*n*=300)	HER2-Positive (*n*=97)	HER2-Low (*n*=155)	HER2-Ultralow (*n*=27)	HER2-Negative (*n*=21)	*p*-value
Nottingham grade						<0.001^b^
Grade 1	49 (16.3)	7 (7.2)	35 (22.6)	5 (18.5)	2 (9.5)	
Grade 2	171 (57.0)	66 (68.0)	86 (55.5)	11 (40.7)	8 (38.1)	
Grade 3	80 (26.7)	24 (24.7)	34 (21.9)	11 (40.7)	11 (52.4)	
Necrosis present	114 (38.0)	44 (45.4)	53 (34.2)	9 (33.3)	8 (38.1)	0.330^b^
LVI present	80 (26.7)	26 (26.8)	42 (27.1)	9 (33.3)	3 (14.3)	0.517^b^
PNI present	18 (6.0)	3 (3.1)	13 (8.4)	1 (3.7)	1 (4.8)	0.344^b^
sTILs %, median (IQR)	15 (7–30)	20 (7–30)	15 (7–30)	20 (12–30)	20 (7–30)	0.135^c^
ER positive	202 (67.3)	62 (63.9)	116 (74.8)	16 (59.3)	8 (38.1)	**0.004** ^b^
PR positive	149 (49.7)	34 (35.1)	95 (61.3)	15 (55.6)	5 (23.8)	**<0.001** ^b^
Ki-67%, median (IQR)	40 (20–60)	40 (30–60)	32 (16–58)	38 (20–68)	45 (38–60)	0.057^c^
Ki-67 category						**0.004** ^b^
Low (<20%)	63 (21.0)	11 (11.3)	45 (29.0)	5 (18.5)	2 (9.5)	
High (≥20%)	237 (79.0)	86 (88.7)	110 (71.0)	22 (81.5)	19 (90.5)	
Molecular subtype						**<0.001** ^b^
Luminal A	37 (12.3)	6 (6.2)	26 (16.8)	3 (11.1)	2 (9.5)	
Luminal B	167 (55.7)	57 (58.8)	90 (58.1)	13 (48.1)	7 (33.3)	
HER2 Enriched	34 (11.3)	34 (35.1)	0 (0.0)	0 (0.0)	0 (0.0)	
Triple Negative	62 (20.7)	0 (0.0)	39 (25.2)	11 (40.7)	12 (57.1)	

All *p*-values in this table represent omnibus comparisons across all four HER2 expression subgroups simultaneously (HER2-Positive vs. HER2-Low vs. HER2-Ultralow vs. HER2-Negative); no pairwise post-hoc comparisons are presented

Cramer’s V effect sizes for significant associations: Nottingham grade 0.194; ER 0.212; PR 0.275; Ki-67 category 0.210; molecular subtype 0.365

*LVI* lymphovascular invasion, *PNI* perineural invasion, *sTILs* stromal tumour-infiltrating lymphocytes

^b^Pearson’s chi-square test; Fisher’s exact test was substituted when more than 20% of expected cell counts fell below 5. ^c^Kruskal-Wallis H test

Significant *p*-values are highlighted in bold

**Table 3:** Spearman rank correlations among continuous variables

Variable	Spearman ρ	*p*-value	*n*	Strength
Ki-67% vs. sTILs %	0.263	**< 0.001**	300	Weak positive
Age vs. Ki-67%	−0.151	**0.009**	300	Weak negative
Age vs. sTILs %	−0.032	0.579	300	Negligible

Two-tailed Spearman rank correlation coefficients. Strength: |ρ| < 0.1 negligible; 0.1–0.3 weak; 0.3–0.5 moderate; 0.5–0.7 strong; >0.7 very strong

Significant *p*-values are highlighted in bold

**Fig. 2:**
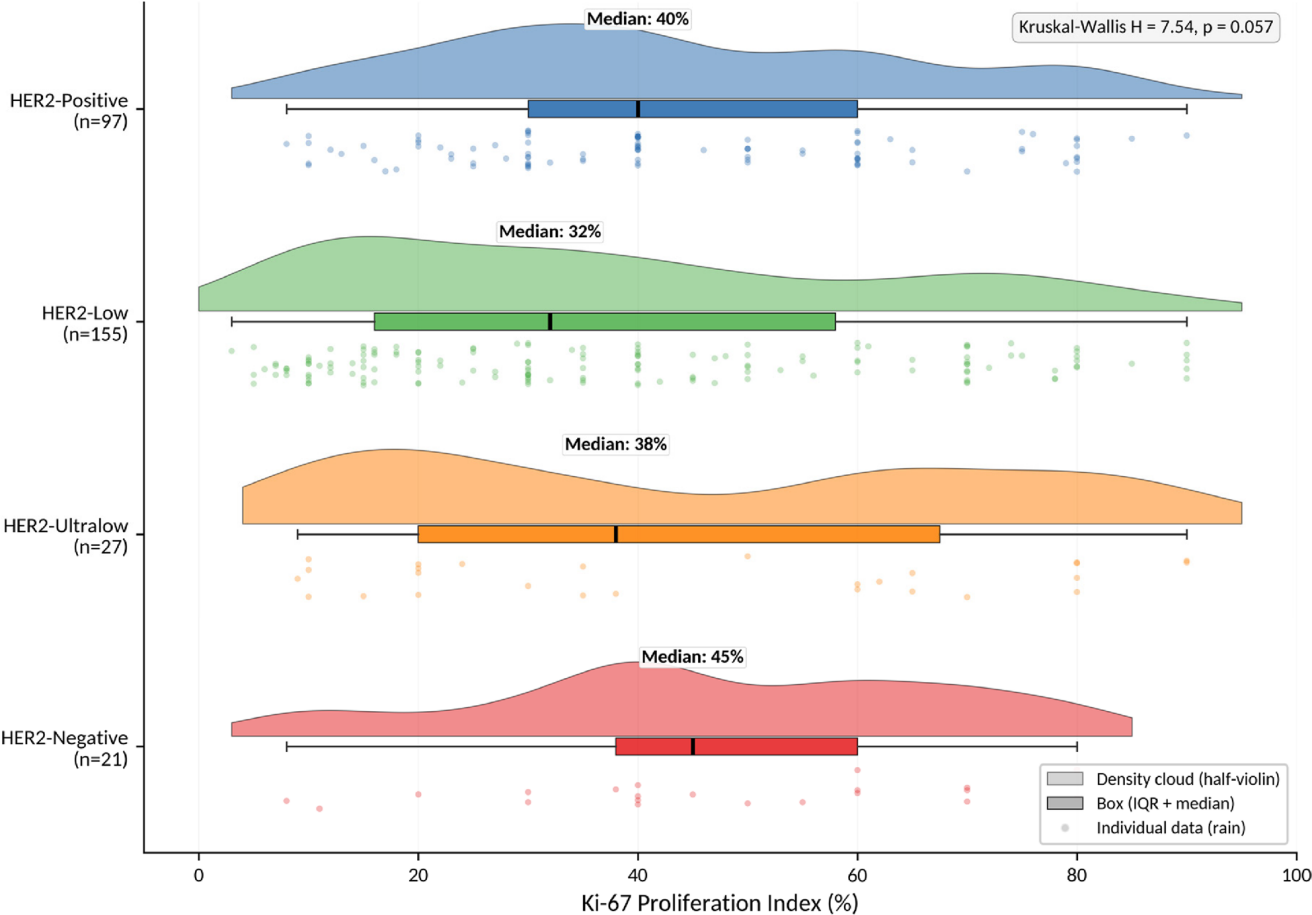
Raincloud plot of Ki-67 proliferation index across HER2 expression subgroups in 300 IBC-NST cases, combining density estimation, summary statistics, and individual data points. Each subgroup is represented by three complementary visualisation layers arranged horizontally. Upper layer: half-violin kernel density estimate (probability cloud) showing distributional shape. Middle layer: box plot displaying median (thick black line), interquartile range (coloured box), and whiskers extending to 1.5 times the IQR. Lower layer: jittered individual data points (rain). Subgroup colours: blue = HER2-Positive (*n* = 97), green = HER2-Low (*n* = 155), orange = HER2-Ultralow (*n* = 27), red = HER2-Negative (*n* = 21). Median values are annotated above each cloud. The Kruskal-Wallis H statistic and p-value are displayed in the upper-right corner

The Ki-67 proliferation index showed a borderline four-group difference when analysed as a continuous variable (Kruskal-Wallis H = 7.54, *p* = 0.057). However, distributional profiles differed substantially (Fig. 2): HER2-low tumours displayed a right-skewed density concentrated at lower values (median 32%, IQR: 16–58%), while HER2-negative tumours showed a near-symmetric distribution centred at higher values (median 45%, IQR: 38–60). The Kolmogorov-Smirnov test confirmed significant distributional differences between HER2-low and HER2-positive (D = 0.190, *p* = 0.023) and between HER2-low and HER2-negative (D = 0.317, *p* = 0.038). When categorized at 20% threshold, the proportion of low Ki-67 was significantly higher in HER2-low (29.0%) compared to HER2-positive (11.3%) and HER2-negative (9.5%; χ² = 13.25, *p* = 0.004, V = 0.210). Stromal TILs did not differ significantly across the four groups (Kruskal-Wallis H = 5.55, *p* = 0.135), though a significant positive Spearman correlation was observed between Ki-67 and sTILs across the entire cohort (ρ = 0.263, *p* < 0.001, *n* = 300).

**Fig. 3:**
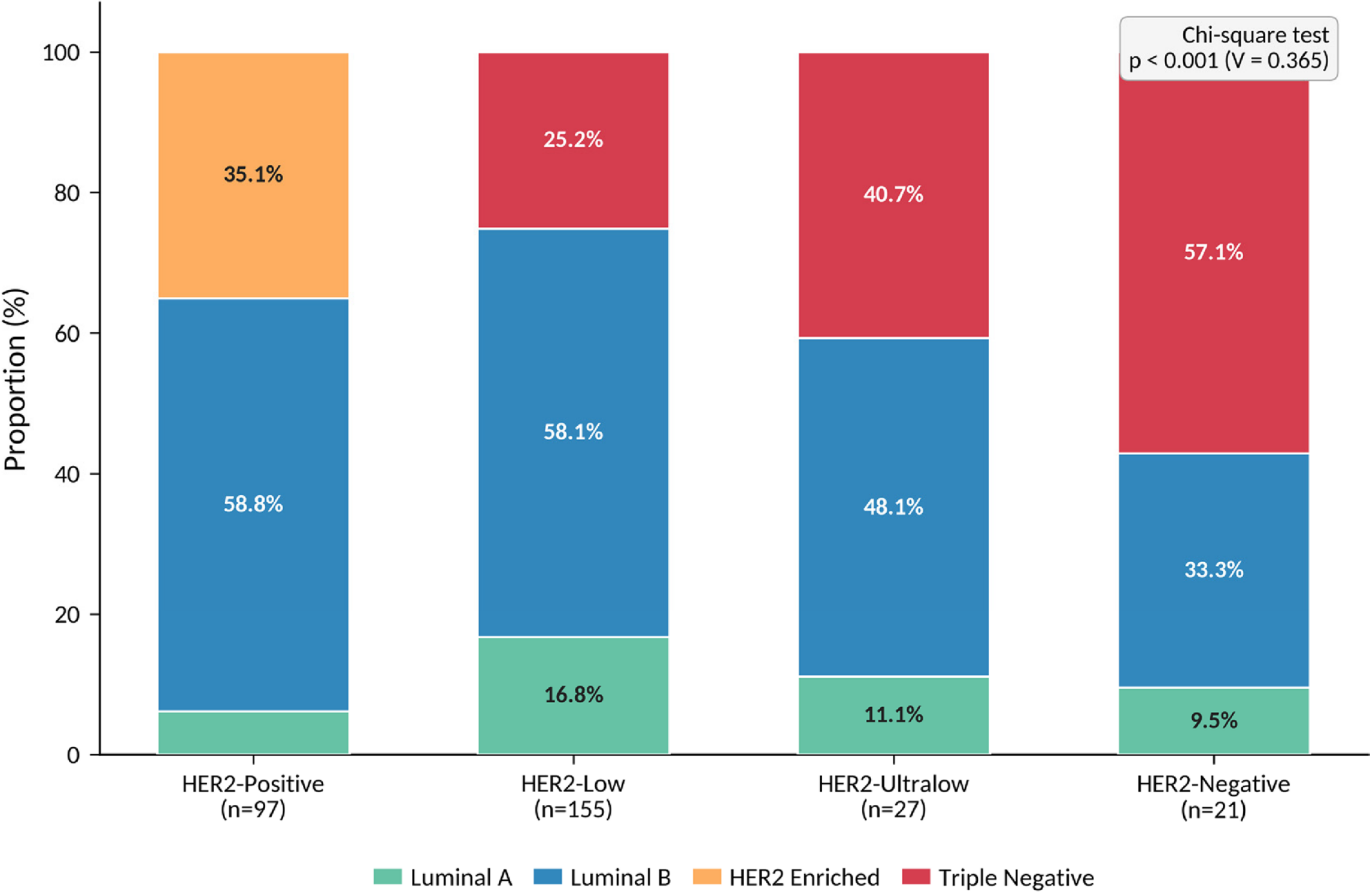
Distribution of molecular subtypes across HER2 expression subgroups in 300 IBC-NST cases, displayed as 100% stacked proportional bar chart. Each bar represents one HER2 subgroup, with segments showing the proportional representation of Luminal A (light blue), Luminal B (blue), HER2-Enriched (orange), and Triple Negative (red) molecular subtypes. Percentages are annotated within each segment. The chi-square statistic, degrees of freedom, p-value, and Cramer’s V effect size are shown below the chart

Molecular subtype distribution was strongly associated with HER2 subgroup (χ² = 119.6, *p* < 0.001, V = 0.365; Fig. 3). Luminal B predominated across HER2-positive (58.8%), HER2-low (58.1%), and HER2-ultralow (48.1%) groups. HER2-enriched subtype was exclusive to HER2-positive tumours (35.1%), with no HER2-enriched cases in the remaining three subgroups. Triple-negative subtype increased progressively from HER2-low (25.2%) through HER2-ultralow (40.7%) to HER2-negative (57.1%).

**Fig. 4:**
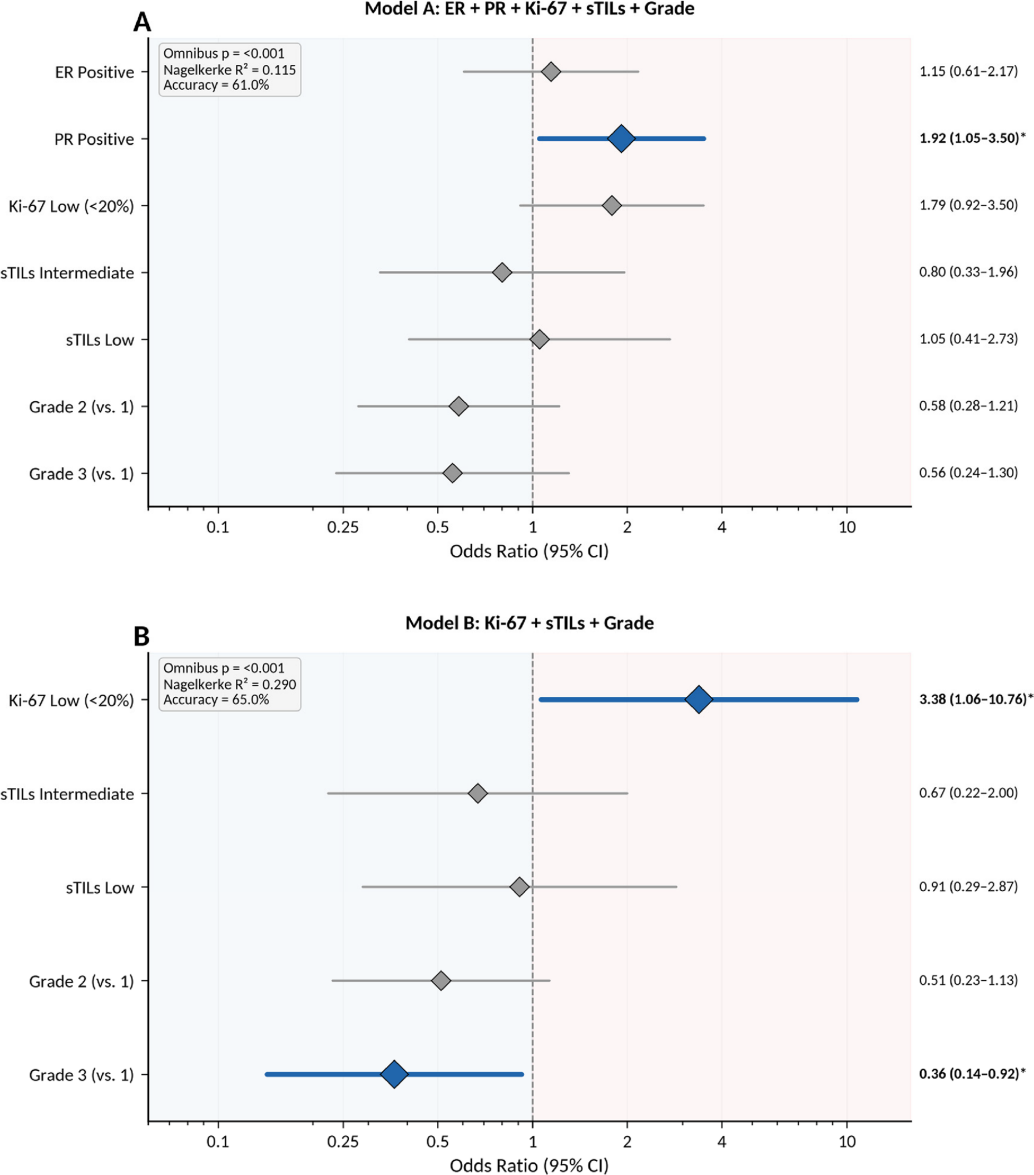
Forest plot of binary logistic regression analysis for independent predictors of HER2-Low status (outcome: HER2-Low = 1 versus all other subgroups = 0) in 300 IBC-NST cases. Odds ratios (OR) with 95% confidence intervals are displayed on a logarithmic x-axis. Blue diamonds indicate statistically significant predictors (*p* < 0.05); grey diamonds indicate non-significant predictors. The dashed vertical line at OR = 1.0 represents the null effect. **A** Model A: individual biomarkers (ER, PR, Ki-67 category, Nottingham grade). **B** Model B: molecular classification (Ki-67 category, Nottingham grade; molecular subtype dummies not displayed due to quasi-complete separation). Model fit statistics are annotated below each panel. OR, odds ratio; CI, confidence interval. * *p* < 0.05

Binary logistic regression identified independent predictors of HER2-low status (Table 4; Fig. 4). In a model including ER, PR, Ki-67 category, sTILs category, and Nottingham grade (omnibus χ² = 26.95, *p* < 0.001; Hosmer-Lemeshow *p* = 0.898; Nagelkerke R² = 0.115), PR-positive status was the sole significant independent predictor (OR = 1.918, 95% CI: 1.052–3.497, *p* = 0.034). In a complementary model excluding molecular subtype to avoid tautology (omnibus χ² = 73.60, *p* < 0.001; R² = 0.290), low Ki-67 (OR = 3.385, 95% CI: 1.065–10.760, *p* = 0.039) and grade 3 (inverse; OR = 0.363, 95% CI: 0.143–0.923, *p* = 0.033) emerged as significant predictors.

**Table 4:** Binary logistic regression: independent predictors of HER2-Low status (HER2-Low vs. all others)

Predictor	Wald χ²	*p*-value	OR	95% CI
Model A: Individual biomarkers^a^				
PR positive (vs. negative)	4.515	**0.034**	1.918	1.052–3.497
ER positive (vs. negative)	0.173	0.677	1.145	0.605–2.166
Ki-67 low < 20% (vs. high)	2.893	0.089	1.789	0.915–3.499
Grade 2 (vs. grade 1)	2.075	0.150	0.583	0.280–1.215
Grade 3 (vs. grade 1)	1.820	0.177	0.557	0.238–1.304
Model B: Molecular classification^b^				
Ki-67 low < 20% (vs. high)	4.269	**0.039**	3.385	1.065–10.760
Grade 3 (vs. grade 1)	4.526	**0.033**	0.363	0.143–0.923
Grade 2 (vs. grade 1)	2.730	0.099	0.512	0.231–1.133

^a^Model A: ER + PR + Ki-67 category + sTILs category + Nottingham grade (sTILs categories not significant, not shown). Omnibus χ² = 26.950 (df = 7, *p* < 0.001); Hosmer–Lemeshow χ² = 3.517 (*p* = 0.898, indicating good fit); Nagelkerke R² = 0.115; classification accuracy = 61.0%. ^b^Model B: Molecular subtype + Ki-67 category + sTILs category + Nottingham grade (molecular subtype dummies and sTILs not shown due to quasi-separation/non-significance). Omnibus χ² = 73.601 (df = 8, *p* < 0.001); Hosmer–Lemeshow χ² = 9.065 (*p* = 0.248); Nagelkerke R² = 0.290; classification accuracy = 65.0%. *n* = 300. *OR* odds ratio, *CI* confidence interval

Significant *p*-values are highlighted in bold

**Fig. 5:**
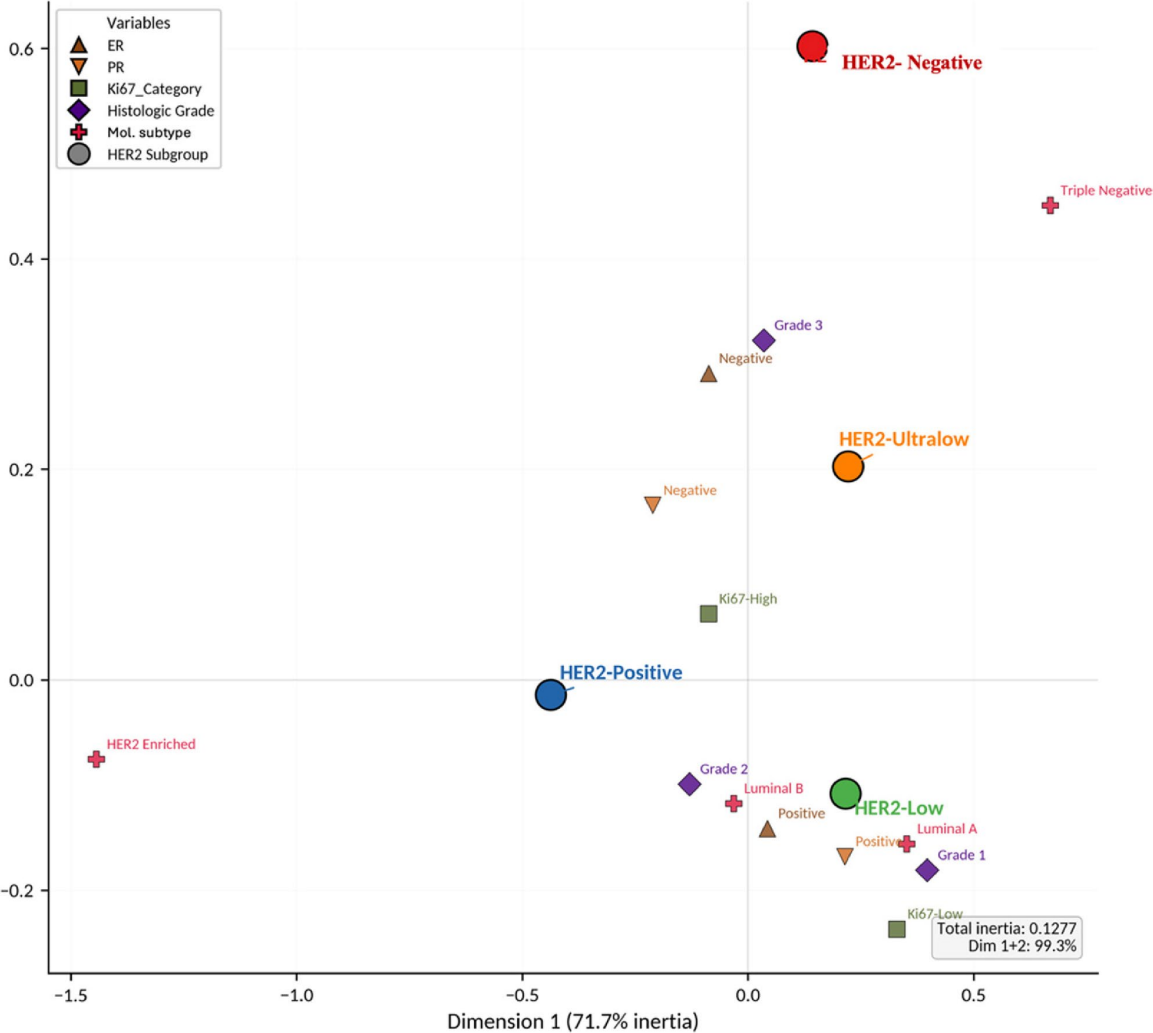
Correspondence analysis biplot mapping the multivariate associations between HER2 expression subgroups and clinicopathologic features in 300 IBC-NST cases. Biplot derived from correspondence analysis of the contingency table crossing four HER2 subgroups (row points, displayed as large labelled circles) against categories of five variables: ER status, PR status, Ki-67 category (< 20% vs. ≥ 20%), Nottingham grade (1, 2, 3), and molecular subtype (column points, displayed as small markers coded by variable type). Proximity between points indicates association; opposing positions indicate inverse relationships. Dimension 1 (horizontal axis) and Dimension 2 (vertical axis) are labelled with their respective percentage contributions to total inertia (total inertia = 0.394)

**Fig. 6:**
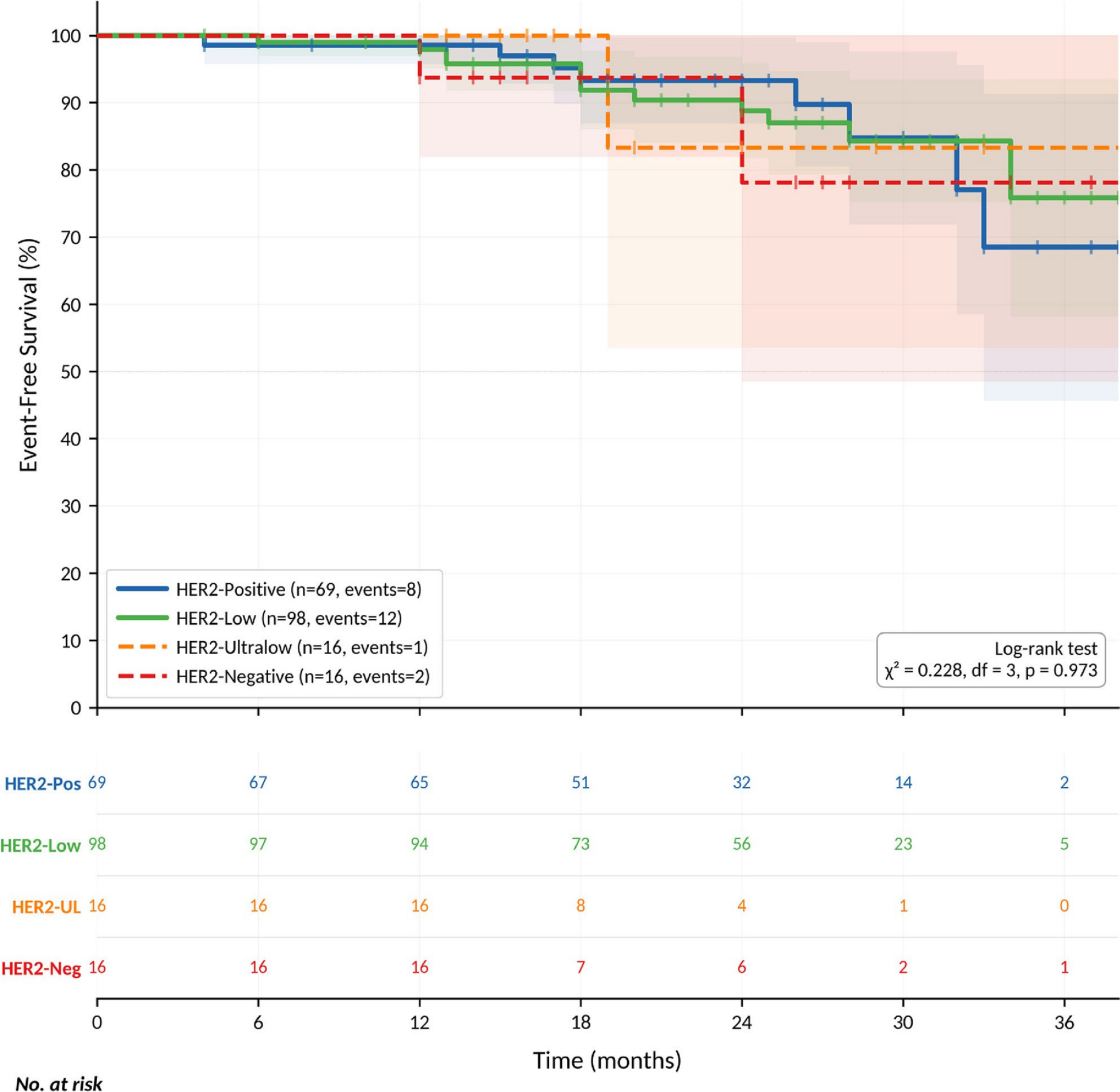
Kaplan-Meier event-free survival curves stratified by HER2 expression subgroup in 199 IBC-NST patients with available follow-up data. Events were defined as disease recurrence, relapse, or death from a related cause; all other patients were censored at last contact. Line styles: HER2-Positive (blue, solid; *n* = 69, 8 events), HER2-Low (green, solid; *n* = 98, 12 events), HER2-Ultralow (orange, dashed; *n* = 16, 1 event), HER2-Negative (red, dashed; *n* = 16, 2 events). Tick marks indicate censored observations. The number-at-risk table below the curve displays patients remaining under observation at 6-month intervals. Log-rank (Mantel-Cox) test statistic and p-value are annotated on the figure

Correspondence analysis simultaneously mapped the joint associations between HER2 subgroups and all five significant categorical variables (Fig. 5), with the first two dimensions capturing 99.3% of total inertia. HER2-low clustered with ER-positive, PR-positive, Luminal A, grade 1, and low Ki-67 features in the lower-right quadrant, while HER2-negative clustered with triple-negative, grade 3, and hormone receptor-negative features in the upper-right. HER2-ultralow occupied an intermediate position between HER2-low and HER2-negative on the second dimension, supporting its characterization as a transitional phenotype. Follow-up data were available for 199 patients (66.3%), with a median of 23.0 months. Neither event-free survival (log-rank χ² = 0.228, *p* = 0.973) nor overall survival (log-rank χ² = 1.158, *p* = 0.763) differed significantly across subgroups (Fig. 6).

## Discussion

The traditional dichotomy of HER2 status in breast cancer has given way to a more granular classification that now includes HER2-low and HER2-ultralow categories. This shift, driven by the efficacy of trastuzumab deruxtecan in the DESTINY-Breast04 trial and its extension to HER2-ultralow disease in DESTINY-Breast06, has made precise characterization of these subgroups essential [6, 7]. This study provides a comprehensive four-group comparison in 300 IBC-NST cases from an Indian tertiary centre, incorporating multivariate analyses seldom applied in this context.

HER2-low constituted 51.7% of cases, consistent with the 45–55% range reported by Schettini et al.., Agostinetto et al.., and Denkert et al. [9, 11, 12]. Among Indian studies, Gogia et al. reported a comparable 59.9% in 1,932 cases, while Melemadathil et al. found a strikingly lower 13.1% in 1,845 cases [13, 14]. These disparities likely reflect differences in IHC platforms, fixation protocols, and interobserver variability in interpreting faint membrane staining, as emphasized by Marchiò et al. [5].

The hormone receptor profile of HER2-low tumours (ER 74.8%, PR 61.3% positivity) aligns with the consistent observation across the literature. Zhang et al. reported 93.1% ER and 85.5% PR positivity, while Schettini et al. (ER 87%, PR 71.8% positivity) and Lu et al. documented comparable proportions [8, 9, 15]. The somewhat lower figures in our cohort may reflect the higher baseline triple-negative rate characteristic of Indian populations [16]. In our exploratory logistic regression, PR-positive status was the sole independent predictor of HER2-low classification after adjusting for ER, Ki-67, sTILs, and grade (OR = 1.918, *p* = 0.034). While higher PR positivity in HER2-low tumours is a consistent observation across the literature, prior studies have reported this association descriptively rather than through multivariate modelling. Schettini et al. and Denkert et al. documented higher PR positivity in HER2-low cohorts but did not employ logistic regression with HER2-low as the outcome [9, 12]. Agostinetto et al. observed a similar gradient but restricted their multivariate analysis to survival endpoints [11], and Zhang et al. described the association within a genomic profiling framework without modelling PR as a classifier [8]. To our knowledge, the present study is among the first to identify PR positivity as an independent predictor of HER2-low status after adjustment for ER and other covariates, though the modest model fit (R² = 0.115) means this finding warrants validation in larger, independent cohorts.

Whether the observed PR gradient reflects biology or technical artifact deserves consideration. PR immunohistochemistry is more vulnerable to pre-analytical degradation than ER, with prolonged cold ischaemia and delayed fixation preferentially reducing PR expression. However, a uniform antibody clone (PgR636) and staining platform were applied across all 300 cases, so any systematic bias would be expected to affect all HER2 subgroups equally rather than selectively enrich PR positivity in HER2-low tumours. The PR gradient also tracks coherently with ER status and molecular subtype distribution, and its direction is consistent with findings from independent groups using different platforms and clones, arguing against a site-specific artifact. Confirmation through alternative antibody clones or mRNA-level quantification would nevertheless strengthen this interpretation.

Nottingham grade distribution followed expected patterns: HER2-low was predominantly grade 2 (55.5%), consistent with Abbasvandi et al. and Tarantino et al. [17, 18]. The high grade 3 proportion in HER2-negative (52.4%) and HER2-ultralow (40.7%) distinguishes these groups from HER2-low. Ordinal logistic regression confirmed Ki-67 as the strongest grade predictor at both thresholds (grade 1 vs. 2 + 3: OR = 0.242, *p* < 0.001; grade 1 + 2 vs. 3: OR = 0.145, *p* = 0.002), while HER2 subgroup was not independently predictive, indicating that grade differences are mediated through the biomarker profile rather than HER2 expression itself.

The Ki-67 analysis illustrates how complementary approaches extract signal that standard tests miss. The four-group Kruskal-Wallis was borderline (*p* = 0.057), yet the categorical comparison at 20% was clearly significant (*p* = 0.004), and Kolmogorov-Smirnov testing confirmed genuine distributional shape differences between HER2-low and both HER2-positive (*p* = 0.023) and HER2-negative (*p* = 0.038). Distributional analysis revealed that HER2-low Ki-67 is right-skewed (skewness = 0.52), reflecting enrichment of low-proliferating tumours, concordant with Zhang et al. and Li et al. [8, 19]. The discordance with Gamrani et al. who reported higher Ki-67 in HER2-low, likely reflects population differences and scoring methodology [20].

The Sankey diagram (Fig. 1) highlights the compositional heterogeneity of HER2-low: 63.9% derive from IHC 1 + and 36.1% from IHC 2+/FISH non-amplified. IHC 2 + emerges as the critical node where 39.8% are reclassified as HER2-positive after FISH, reinforcing the 2023 ASCO-CAP recommendation for mandatory FISH reflex testing [4]. The exclusive mapping of HER2-enriched to HER2-positive and the progressive triple-negative gradient confirm that these subgroups occupy distinct molecular positions, concordant with Ahuja et al. and Schettini et al. [9, 21].

The correspondence analysis biplot (Fig. 5) helps to show that HER2-ultralow’s intermediate position on Dimension 2 supports its characterization as a transitional phenotype, as suggested by Shi et al. and Franchina et al. [22, 23].

No significant survival differences were observed, consistent with Mutai et al.., Horisawa et al., and Agostinetto et al. [11, 24, 25] Even in larger cohorts, the prognostic impact has been inconsistent with Tan et al. and Molinelli et al. reporting advantages for HER2-low, while Hein et al. found none [26–28]. Current evidence suggests that HER2-low’s clinical significance lies primarily in predicting Antibody Drug Conjugate (ADC) response rather than natural history prognostication.

The weak but significant positive correlation between Ki-67 and sTILs (ρ = 0.263, *p* < 0.001) adds a tumour microenvironment dimension to the characterization. This biologically coherent finding, suggesting that rapidly proliferating tumours generate more neoantigens and recruit more immune cells, was also observed by Lu et al., who reported higher mean sTILs in HER2-positive than HER2-low tumours [15]. The negative correlation between age and Ki-67 (ρ = −0.151, *p* = 0.009) further supports the picture of HER2-low as a tumour of slightly older patients with lower proliferation and less immune engagement, a biologically coherent luminal phenotype.

If the proportion of HER2-low cases observed in this single-centre cohort (51.7%) is broadly representative of the Indian population, the absolute number of patients potentially eligible for ADC therapy would be considerable. Our data show that IHC 1 + cases account for nearly two-thirds of HER2-low tumours, and this is the scoring category where diagnostic subjectivity is most pronounced. Multicentre validation of these proportions, combined with standardized reporting templates and digital pathology tools, will be needed to establish reliable estimates of ADC-eligible populations in India.

The analytical depth of this study represents a methodological advance over most single-centre HER2-low publications, which typically rely exclusively on bivariate testing. The identification of PR as an independent predictor through logistic regression, the demonstration of Ki-67 distributional shape differences through Kolmogorov-Smirnov testing, and the multivariate mapping through correspondence analysis provide a layered characterization that captures associations invisible to standard crosstabulations. These approaches are particularly relevant given the ongoing debate about whether HER2-low represents a distinct biological entity or merely the middle of a continuous HER2 expression spectrum. Our data, particularly the correspondence analysis showing clear spatial separation of all four subgroups with 99.3% inertia captured in two dimensions, strongly favour the former interpretation.

A distinguishing feature of this study is the inclusion of HER2-ultralow as a separate analytical category, a decision rooted in the evolving therapeutic landscape. The DESTINY-Breast06 trial demonstrated T-DXd efficacy in HER2-ultralow metastatic breast cancer, expanding the treatment-eligible population beyond HER2-low. Our data show that HER2-ultralow occupies an intermediate position across multiple parameters: its ER positivity (59.3%) falls between HER2-low (74.8%) and HER2-negative (38.1%), and its molecular subtype distribution straddles luminal and triple-negative profiles. This intermediate phenotype, corroborated by the correspondence analysis, supports the biological rationale for treating HER2-ultralow as a separate entity rather than grouping it with HER2-negative—a distinction with direct therapeutic implications as ADC access expands.

### Limitations of the study

Several limitations warrant explicit acknowledgement. This is a single-centre retrospective study conducted at a tertiary academic institution, and the findings may not be generalizable to community practice settings, other populations, or alternative IHC platforms and antibody clones. The smaller subgroups, HER2-ultralow (*n* = 27) and HER2-negative (*n* = 21), constrain statistical power and estimate precision. Although the three-group sensitivity analysis confirmed that the principal findings are robust, some comparisons involving these subgroups (particularly the pairwise KS tests and Fisher’s exact tests) should be interpreted with caution. The distinction between HER2-ultralow and HER2-negative rests on one of the most subjective assessments in surgical pathology, the presence versus absence of barely perceptible membrane staining. Although two pathologists reviewed all IHC 0 cases with consensus resolution of discordant interpretations, no formal interobserver agreement statistic was calculated. Published concordance rates for this distinction are as low as 57%, and misclassification at this threshold directly affects the four-group analysis. Additionally, the HER2 classifications were based solely on interpretation of core biopsy samples. Several recent studies have demonstrated that HER2 status shows appreciable discordance between core biopsy and matched excision specimens especially when the HER2-low and HER2-zero categories are considered separately [29, 30]. In the present study, patients did not have their biopsy and excision HER2 scores formally compared, and this may have attenuated or inflated some of the observed differences.

The survival analysis is constrained by the short median follow-up (23 months) and the very small number of events (17 recurrences, 6 deaths among 199 patients with follow-up). Breast cancer survival studies, particularly for hormone receptor-positive disease, typically require 5–10 years of follow-up to detect meaningful differences and hence no prognostic conclusions can be drawn from these data. BRCA mutation status and genomic profiling data (such as Oncotype DX or MammaPrint) were unavailable, limiting the depth of biological characterization possible in this retrospective design. The Ki-67 assessment remains semi-quantitative with inherent interobserver and interlaboratory variability, and the observed differences between our results and those of other studies may partly reflect methodological heterogeneity in cutoff selection, counting methods, and hot-spot versus global assessment strategies. The same consideration applies to sTILs evaluation, which was performed according to the International TILs Working Group recommendations but whose reproducibility could not be formally quantified.

## Conclusion

This study provides a four-group clinicopathologic characterization of 300 IBC-NST cases across the HER2 expression spectrum. Demographic characteristics, clinical stage, tumour size, lymphovascular invasion, perineural invasion, and stromal tumour-infiltrating lymphocyte density did not differ across the four subgroups, indicating that these parameters do not discriminate between HER2-defined categories in this cohort. HER2-low tumours, constituting 51.7% of cases, were associated with higher hormone receptor positivity, lower histologic grade, and a greater proportion of low-proliferative tumours compared to the other subgroups. In exploratory logistic regression, PR-positive status emerged as the only individual biomarker independently associated with HER2-low classification, though the model’s modest explanatory power means this finding requires independent confirmation. Correspondence analysis provided visual evidence for the multivariate biological structuring of all four subgroups, with HER2-ultralow occupying an intermediate position between HER2-low and HER2-negative. Survival did not differ across subgroups, though the limited event count precludes meaningful prognostic interpretation. HER2 staining heterogeneity was more frequent in HER2-low than HER2-positive tumours, with implications for diagnostic reproducibility on biopsy specimens. These findings support the clinical utility of distinguishing HER2-low and HER2-ultralow categories in pathology reporting and reinforce the need for standardized IHC interpretation at the lower end of the HER2 expression spectrum. Prospective multicentre studies with formal interobserver reproducibility assessment and extended follow-up are warranted to validate these observations.

## Data Availability

The datasets generated and analysed during the current study are not publicly available, as they are derived from patient medical records held under institutional governance and public archiving would risk compromise of individual patient privacy. The datasets are available from the corresponding author on reasonable request, subject to institutional data governance approval from Manipal Academy of Higher Education, Manipal, India.
